# 

**Published:** 1894-11

**Authors:** 


					﻿Vol. XIV.
NOVEMBER, 1894.
No 11.
THE
pOMIOPATHlC pHY^lClAJl,
A Monthly Journal of Homoeopathic Materia Medica
and Clinical Medicine.
“ He ia a freeman whom truth makea free, and all are alavee betide."
“Beek the Truth: come whence it may, coat what it will."
EDITED AND PUBLISH ED BY
WALTER M. JAMES, M. D.
PHILADELPHIA :
ZETO. 1231 LOCUST STREET.
LONDON:
ALFRED HEATH & CO., 8olb Agents bob Great Britain,
114 Ebury 8treet, Eaton Square.
Yearly Subscription, $2.50, invariably in advance. Single numbers, 25 etc.
Entered at the Philadelphia Peat-Offiee aa Baeend Claea Mali Matter,
				

